# Decreased duration of mechanical ventilation when comparing analgesia-based sedation using remifentanil with standard hypnotic-based sedation for up to 10 days in intensive care unit patients: a randomised trial [ISRCTN47583497]

**DOI:** 10.1186/cc3495

**Published:** 2005-03-15

**Authors:** Des Breen, Andreas Karabinis, Manu Malbrain, Rex Morais, Sven Albrecht, Inge-Lise Jarnvig, Pauline Parkinson, Andrew JT Kirkham

**Affiliations:** 1 Consultant in Anaesthesia and Intensive Care, Royal Hallamshire Hospital, Sheffield, UK; 2 Director of Intensive Care Unit, Genimatas General Hospital, Athens, Greece; 3 Director of Intensive Care Unit, ZiekenhuisNetwerk Antwerpen, Antwerpen, Belgium; 4 Consultant Anaesthetist, Intensive Care Unit, Dubai Hospital, Dubai, United Arab Emirates; 5 Deputy Director, Universität Erlangen-Nürnberg, Klinik für Anästhesiologie, Erlangen, Germany; 6 Senior Registrar, Intensive Care Unit, Righospitalet, Copenhagan, Denmark; 7 Clinical Scientist, Neurosciences Medicines Development Centre, GlaxoSmithKline, Greenford, Middlesex, UK; 8 Clinical Development Director, Neurosciences Medicines Development Centre, GlaxoSmithKline, Greenford, Middlesex, UK

## Abstract

**Introduction:**

This randomised, open-label, multicentre study compared the safety and efficacy of an analgesia-based sedation regime using remifentanil with a conventional hypnotic-based sedation regime in critically ill patients requiring prolonged mechanical ventilation for up to 10 days.

**Methods:**

One hundred and five randomised patients received either a remifentanil-based sedation regime (initial dose 6 to 9 μg kg^-1 ^h^-1 ^(0.1 to 0.15 μg kg^-1 ^min^-1^) titrated to response before the addition of midazolam for further sedation (*n *= 57), or a midazolam-based sedation regime with fentanyl or morphine added for analgesia (*n *= 48). Patients were sedated to an optimal Sedation–Agitation Scale (SAS) score of 3 or 4 and a pain intensity (PI) score of 1 or 2.

**Results:**

The remifentanil-based sedation regime significantly reduced the duration of mechanical ventilation by more than 2 days (53.5 hours, *P *= 0.033), and significantly reduced the time from the start of the weaning process to extubation by more than 1 day (26.6 hours, *P *< 0.001). There was a trend towards shortening the stay in the intensive care unit (ICU) by 1 day. The median time of optimal SAS and PI was the same in both groups. There was a significant difference in the median time to offset of pharmacodynamic effects when discontinuing study medication in patients not extubated at 10 days (remifentanil 0.250 hour, comparator 1.167 hours; *P *< 0.001). Of the patients treated with remifentanil, 26% did not receive any midazolam during the study. In those patients that did receive midazolam, the use of remifentanil considerably reduced the total dose of midazolam required. Between days 3 and 10 the weighted mean infusion rate of remifentanil remained constant with no evidence of accumulation or of a development of tolerance to remifentanil. There was no difference between the groups in SAS or PI score in the 24 hours after stopping the study medication. Remifentanil was well tolerated.

**Conclusion:**

Analgesia-based sedation with remifentanil was well tolerated; it reduces the duration of mechanical ventilation and improves the weaning process compared with standard hypnotic-based sedation regimes in ICU patients requiring long-term ventilation for up to 10 days.

## Introduction

Most patients who require intensive care need effective analgesia and sedation to control potentially unpleasant symptoms such as pain and anxiety. Analgesics and sedatives are also used to allow patients to tolerate nursing procedures and tracheal intubation and to aid in mechanical ventilation. Most commonly, the combined use of a sedative agent with an opioid is used to achieve this. The sedative agent is titrated towards the degree of sedation and opioids are added as judged necessary for pain control.

The pharmacodynamic effects of traditionally used sedative and opioid drugs are unpredictable and often prolonged in the critically ill patient for various reasons. The pharmacokinetics are altered with different volumes of distribution and elimination half-lives. Multiple drugs are given in large doses for prolonged periods, which can lead to drug interactions and tolerance. There is altered organ function with impaired renal/hepatic function, altered regional blood flow, protein and enzyme system dysfunction and altered receptor response. In addition, the most commonly used sedatives and opioids have a context-sensitive half-time that increases with time. Thus, these drugs will accumulate during prolonged use. All opioids have sedative properties to various degrees at high doses. However, traditionally the opioid part of a sedation regime is kept to a minimum to protect against opioid accumulation and an unpredictable recovery/weaning from mechanical ventilation.

Remifentanil hydrochloride is a potent, selective μ-opioid receptor agonist that has a rapid onset of action (about 1 min) and quickly achieves steady state [1]. Remifentanil is metabolised by non-specific plasma esterases and is therefore independent of organ function [2]. Remifentanil is rapidly metabolised and has a context-sensitive half-time of about 2 to 3 min that is independent of duration of infusion [3]. These features of remifentanil make it an ideal agent for use in critically ill patients. It is easy to titrate and can be given in relatively high doses for prolonged periods without risk of accumulation [4,5] or delayed offset of effects [5]. It allows the opioid to be used as the main drug to provide patient comfort with the sedative agent being kept to a minimum. Remifentanil as part of an analgesic-based sedative regime (analgo-sedation) has been studied in critically ill patients for up to 5 days [4-17].

This present study was conducted to assess the efficacy and safety of a prolonged infusion of remifentanil in critically ill patients for up to 10 days in comparison with a standard sedative regime of midazolam plus a traditional opioid. The efficacy of remifentanil was assessed by the primary endpoint of time from starting the study drug until time of extubation. The safety profile of remifentanil was assessed by monitoring haemodynamic parameters and recording adverse events throughout the study period.

## Materials and methods

This study was a randomised, open-label, multicentre, parallel-group study comparing the safety and efficacy of an analgesia-based regime using remifentanil with a conventional hypnotic-based regime in critically ill patients requiring mechanical ventilation for 3 to 10 days. The study was conducted in accordance with good clinical practice and within the guidelines set out in the Declaration of Helsinki. Informed consent/assent was obtained from all patients or their representatives. After approval from local and national ethics committees, 105 patients from 15 centres in 10 countries were recruited. Patients were randomised in a 1:1 ratio to receive either a remifentanil-based regime or a comparator hypnotic-based regime using midazolam with either morphine or fentanyl for analgesia.

### Inclusion and exclusion criteria

The target population were those patients requiring long-term mechanical ventilation for medical reasons. Post-surgical patients requiring extended mechanical ventilation as a result of post-surgical complications were also included. Patients were eligible if they were more than 18 years old, had been admitted to the intensive care unit (ICU) within the previous 30 hours, were expected to require mechanical ventilation for longer than 96 hours and required analgesia and sedation. Females were eligible to enter the study if they were of non-childbearing potential or had a negative pregnancy test at screening and agreed not to fall pregnant for 12 days after stopping the study drug.

Patients were excluded from the study if their medical condition prevented assessment of depth of sedation, if it required the frequent down-titration of analgesics/sedatives for assessment, if it was likely to require surgery or tracheostomy during the treatment period, if it required neuromuscular blocking drugs by infusion, if it required epidural blockade, if it required sedatives or anaesthetic agents other than study drugs specified in the treatment period, or if there was a contraindication to the use of remifentanil, morphine, fentanyl or midazolam. Other exclusions were sensitivity to the drugs or class of drugs specified in the study, a history of alcohol or drug abuse, a concurrent or previous entry into this or other investigational drug studies within 30 days, or pregnancy or lactation. Protocol-specified treatment regimes had to be appropriate for the management of the patients. After 30 patients had entered the study, a protocol amendment allowed the inclusion of patients who had been receiving mechanical ventilation for up to 30 hours irrespective of the time in the ICU, allowed the inclusion of patients requiring surgery of less than 6 hours' duration during the treatment period and reduced the required duration of mechanical ventilation from 96 to 72 hours.

### Study period

The study was divided into four periods: screening, treatment, post-treatment and follow-up.

### Screening period

The screening period was from ICU admission to the start of the study drug and included the time for considering eligibility, obtaining consent/assent, randomisation and assessment of the patient's SAPS II score. Baseline demographics, physiological variables, Sedation–Agitation Scale (SAS) score and pain intensity (PI) score were also assessed during this time. The SAS is a seven-point scoring system, and a SAS score of 3 or 4 was defined as optimal sedation in this study (see Additional file 1, [18]). The PI score is a six-point score where 1 or 2 represents no pain or mild pain (see Additional file 2). Baseline liver function tests and creatinine clearance were also measured.

### Treatment period

The treatment period was from the start of the study drug to permanent discontinuation of the study drug, or after 10 days of administration, or death, whichever was the sooner. SAS, PI, heart rate (HR) and mean arterial pressure (MAP) were continuously monitored throughout the treatment period and were recorded at the time of each bolus dose and/or change in infusion rate of any of the study drugs. These parameters were recorded again when optimal sedation and pain control had been established or re-established. These variables were also recorded at least every 8 hours in the event of no change in study drug. Study drugs and amount administered were also recorded during the treatment period. Liver function tests and creatinine clearance were assessed daily. Before the start of administration of the study drugs the existing sedative/analgesic regime was discontinued. Patients were then sedated to an optimal SAS and PI score by a remifentanil-based regime or a hypnotic-based regime.

#### Remifentanil-based regime

**Figure 1:**
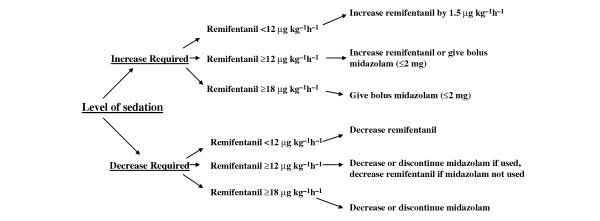
The dosing algorithm.

The remifentanil infusion was started at 6 to 9 μg kg^-1 ^h^-1 ^(0.1 to 0.15 μg kg^-1 ^min^-1^). The remifentanil infusion was titrated in 1.5 μg kg^-1 ^h^-1 ^(0.025 μg kg^-1 ^min^-1^) increments at 5 to 10 min intervals to achieve an optimum level of sedation/analgesia based on clinical judgement. Bolus doses of remifentanil were not permitted. Once the remifentanil infusion reached a rate of 12 μg kg^-1 ^h^-1 ^(0.2 μg kg^-1 ^min^-1^), boluses of midazolam (not more than 2 mg) could be used if required after clinical assessment. Remifentanil was not used as the sole agent for sedation at infusion rates greater than 18 μg kg^-1 ^h^-1 ^(0.3 μg kg^-1 ^min^-1^). Above this rate, midazolam boluses were used. Further increases in the remifentanil rate were allowed for the treatment of pain and in anticipation of short stimulating procedures, up to a maximum rate of 45 μg kg^-1 ^h^-1 ^(0.75 μg kg^-1 ^min^-1^). The remifentanil dosing regime is depicted in Fig. 1[5,10,11].

#### Hypnotic-based regime

Midazolam was used by infusion and/or boluses as the sedative agent, and was titrated to an optimum level of sedation based on clinical judgement and in accordance with standard clinical protocols. Either morphine or fentanyl was used as the analgesic agent, titrated to obtain adequate pain control. The initial dose and subsequent adjustments of sedative and analgesic agents were at the investigators' discretion and in accordance with routine clinical practice to obtain an optimum SAS and PI score.

#### Weaning and extubation

The decision to begin the weaning process was based on clinical judgement and was defined as the time point at which the investigator first adjusted the study drug infusion rate or decided not to give any more boluses of the study drugs so as to encourage spontaneous respiration with the result of extubating the patient. For those patients extubated within 10 days, the study drugs were down-titrated in accordance with clinical judgement until a decision was made to extubate the patient. For patients in the comparator hypnotic-based treatment group this was performed in accordance with routine clinical practice at the investigational site. As a guide, for patients in the remifentanil group who were eligible for extubation, the remifentanil infusion was decreased to 6 μg kg^-1 ^h^-1 ^(0.1 μg kg^-1 ^min^-1^) either immediately or in increments at the investigator's discretion, and no further midazolam boluses were given. Remifentanil was discontinued after extubation and, if necessary, suitable alternative methods of pain relief were instituted.

For patients not extubated within 10 days, the study drugs were discontinued in both groups and the time to offset of pharmacodynamic effects was recorded. As soon as there was a demonstrable change in haemodynamic variables, SAS score or PI score, alternative sedation and analgesic regimes were instituted as soon as clinically indicated.

### Post-treatment period

The post-treatment period was from the end of the treatment period until 24 hours later. MAP, HR, SAS and PI were recorded at 15 min intervals for the first 2 hours, hourly for the next 4 hours then 6-hourly until the end of the post-treatment period.

### Follow-up period

The follow-up period was from the end of the post-treatment period until 6 days later.

### Study endpoints

#### Efficacy

The primary endpoint was the time from the start of study drug to extubation. Secondary endpoints were the time from start of study drug until start of weaning, the time from start of weaning until extubation, the time from start of study drug to ICU discharge, descriptive PI and SAS during the treatment and post-treatment periods, total exposure to study drugs and concomitant sedative requirements.

#### Safety

The safety endpoints were the offset of pharmacodynamic effects of study drugs after permanent discontinuation, haemodynamic effects, clinical adverse events and the requirement for re-intubation. Haemodynamic variables were monitored continuously throughout the study and recorded at the times stated above. Adverse events were recorded from the start of the study drug until the end of the post-treatment period. Serious adverse events were defined as adverse events that resulted in any of the following outcomes: death, life-threatening event, prolongation of hospitalisation, or a disability or incapacity. Important medical events that did not result in death or were not life-threatening were considered serious adverse events when, on the basis of appropriate medical judgement, they jeopardised the patient and required medical or surgical intervention to prevent one of the outcomes listed above. In addition, serious adverse events possibly attributable to study medication were recorded throughout the 6-day follow-up period.

### Statistical analysis

The time to event endpoints were analysed with the generalised Wilcoxon test with a two-sided α level of 5% judged to indicate a statistically significant difference between the treatment groups. The data for patients who did not experience the event were censored in accordance with predetermined rules. The results of these analyses were summarised by using 75th centiles, difference between 75th centiles and its 95% confidence interval because too few patients achieved each event to allow estimates based on median times to be determined with any precision. The confidence intervals were calculated with methods described by Collett [19].

The percentage time of optimal analgesia/sedation was calculated and summarised by the median in each treatment group and the median of all possible differences between the groups and the 95% confidence interval around that difference to give the best estimate of median difference. Treatments were compared by using the Wilcoxon rank sum test.

With the exception of the incidence of re-intubation, no formal statistical analyses were performed on the demographic, baseline or safety data. These data were summarised either by means and standard deviations (SD) or by frequency tables as appropriate to the data.

## Results

Fifty-seven patients were randomised to receive remifentanil and 48 patients to receive comparator. Of the comparator group, 62% (*n *= 30) received midazolam with fentanyl, 15% (*n *= 7) received midazolam with morphine and 23% (*n *= 11) received midazolam alone.

Patient demographics and baseline characteristics are shown in Table 1. The two treatment groups were well matched in terms of patient characteristics and baseline clinical assessments. The time from ICU entry to the start of study medication was slightly longer in the remifentanil group (remifentanil, mean 23.6 hours, median 14.5; comparator, mean 18.9 hours, median 16.9).

### Efficacy

**Figure 2:**
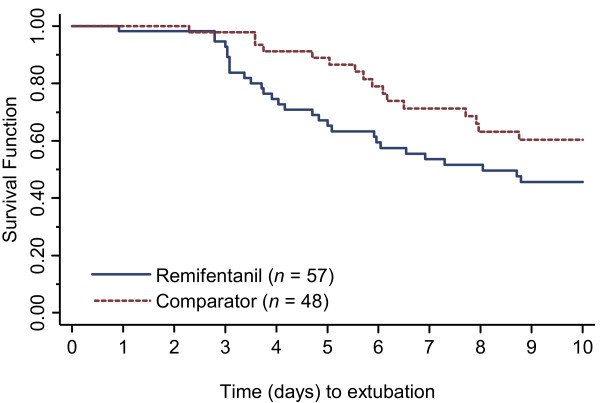
Kaplan–Meier survival plot of time to extubation (days).

Efficacy endpoints are shown in Table 2. Fewer than 50% of patients were extubated during the 10-day treatment period (45 of 105). The 75th centile has been reported for the efficacy endpoints. This analysis records the time at which 25% of all patients reached the assessment points. There was no difference in the time to the start of the weaning process. There was a statistical and clinically significant difference between the two groups in the study's primary endpoint of time of starting the drug to extubation. A Kaplan–Meier plot analysing the duration of mechanical ventilation is shown in Fig. 2. The time difference was 53.5 hours, being shorter in the remifentanil group (*P *= 0.033). The time from the start of the weaning process to extubation was also significantly different at 26.6 hours, also in favour of remifentanil (*P *< 0.001).

The median percentage time of optimal analgesia/sedation was comparable for both groups (remifentanil 96.9%, comparator 97.8%, median difference -0.3, 95% confidence interval -2.7 to 0.2; *P *= 0.16).

**Figure 3:**
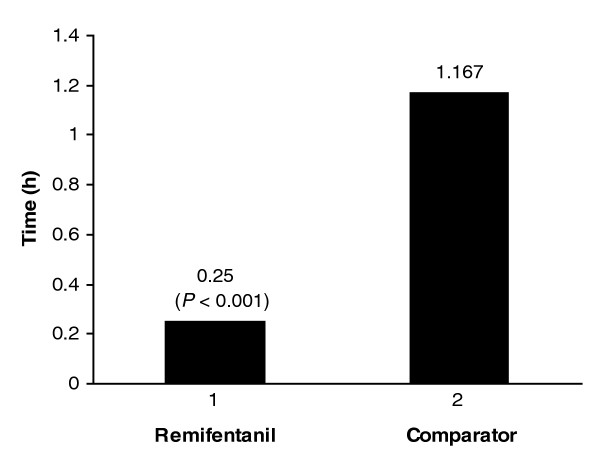
Median time to offset of effects as measured by the time to therapeutic intervention.

There were 16 patients in each group who survived to 10 days but were not extubated, for whom the time to offset of pharmacodynamic effects on discontinuing the study drugs was assessed on 15 patients receiving remifentanil and 14 patients receiving comparator. This was found to be clinically and significantly faster in the remifentanil group (Fig. 3).

### Safety

The incidence of adverse events and the most commonly occurring adverse events (5% or more) is illustrated in Table 3. The profile was similar for the two groups. Liver function tests and creatinine clearance levels were comparable in both groups at baseline and throughout the treatment period. One drug-related serious adverse event was reported. This patient was randomised to receive midazolam and fentanyl. At 1 day after starting study medication the patient developed severe hypotension, which was considered life threatening. There were no reports of muscle rigidity. Comparable MAP and HR values were observed during the post-treatment period.

### Exposure to study drugs

**Figure 4:**
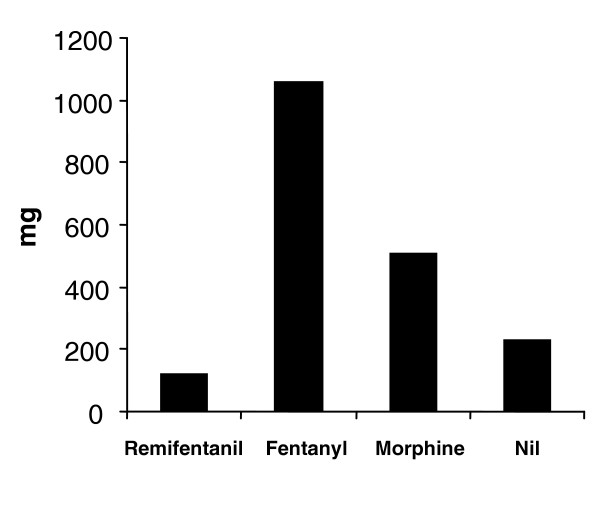
Mean total midazolam dose.

The mean total duration, dose and weighted mean infusion rates of study opioids for all the patients treated in this study are illustrated in Table 4. Patients in the remifentanil group received a longer duration of opioid infusion. Of the patients treated with remifentanil, 26% (15 of 57) did not receive any midazolam during the study. The remaining patients received 1 to 100 boluses during the treatment period. Figure 4 illustrates the mean total midazolam dose in patients receiving opioids and no opioids. There was nearly a ninefold difference in mean total midazolam requirements in the fentanyl group compared with the remifentanil group, and a fourfold difference in the morphine group compared with the remifentanil group.

**Figure 5:**
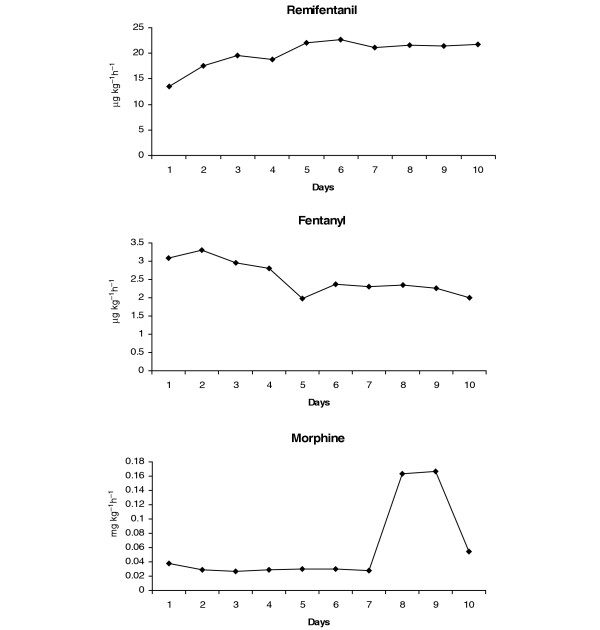
Mean weighted mean opioid infusion over time.

**Figure 6:**
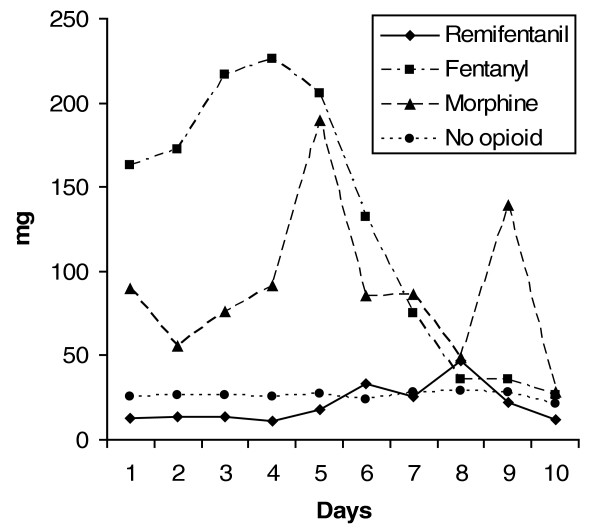
Mean total daily dose of midazolam with concomitant opioids.

Figure 5 shows the weighted mean infusion rate of opioid by day. Remifentanil infusion requirements rose within the first few days then reached a plateau. Figure 6 represents the mean total daily midazolam requirements in combination with each opioid used. The midazolam requirements with remifentanil were the least and did not change significantly with time.

### SAS and PI scores during the post-treatment period

The SAS and PI scores were the same in the remifentanil and comparator groups throughout the post-treatment period. There was no variation over the 24-hour period in either group. The variation in the mean SAS over 24 hours was 3.1 to 3.3 for remifentanil group and 2.7 to 3.0 in the comparator group. The variation in the mean PI over 24 hours was 1.5 to 1.6 for the remifentanil group and 1.5 to 1.7 for the comparator group.

## Discussion

The technique of using remifentanil as the primary sedative and analgesic, with the addition of traditional sedatives such as propofol or midazolam only if necessary, has been studied in ICU patients for up to 3 days [5,11,17] and in neurosurgical patients studied for up to 5 days, with good results [12]. Anecdotally, remifentanil has been used in the ICU population for much longer periods [6]. This is the first study to look at the use of remifentanil for prolonged infusions in ICU patients for up to 10 days. The primary aim of the study was to compare the safety of the techniques and the duration for which patients were on mechanical ventilation.

### Efficacy

The present study has clearly demonstrated a clinically and statistically significant decrease in duration of mechanical ventilation when using remifentanil-based analgesia and sedation. The difference between remifentanil and the comparator group was more than 2 days (53.5 hours). The time from starting the weaning process to extubation was also significantly different by more than 1 day (26.6 hours). Reducing the duration of mechanical ventilation by a matter of days will potentially help to reduce the complications associated with prolonged intubation and ventilation. These differences cannot be explained by differing eligibilities to start the weaning process, because there was no difference between the times to start the weaning process in either of the two study groups. The SAS and PI scores were the same in both groups from baseline through the treatment period and to the end of the post-treatment period. The difference therefore cannot be explained by different sedation levels between the groups.

There are difficulties of comparing extubation times between sedative regimes in an ICU study. Studies have looked at extubation times after anaesthesia, comparing various drugs [20,21]. The time frame for these studies is much shorter, and the times of decisions to stop study drug(s) and extubate patients are much easier to define. It is possible that a critically ill patient will undergo several increases and decreases in sedative and analgesic agents before the decision is made to wean and extubate. It is possible that patients will have been on no drug for some time. Given these points, the difference in the primary endpoint of the study in a small group of patients is remarkable.

There was a trend towards a shorter ICU stay in the remifentanil group by 1 day although this was not statistically significant. This may be because the numbers of patients who were actually extubated within the study period were small and the study was not sufficiently powered to detect the difference. Whereas discharge from an ICU setting is multifactorial and often depends on factors not related to the medical condition of the patient such as bed shortages on the ward and the time of day. The trend in reduced duration of ICU stay is supported by the work of Matthey and colleagues [22], who showed that remifentanil supplemented with propofol significantly reduced the time on mechanical ventilation and allowed earlier discharge from the ICU than an analgesia-based sedation with fentanyl/midazolam. In another study comparing remifentanil/midazolam with morphine/midazolam in a similar way to this study, there was a significant difference between the extubation times and ICU discharge times between the two groups [17]. However, the duration of mechanical ventilation was relatively short.

The context-sensitive half-time of remifentanil is constant and independent of duration of drug administration when administered for up to 6 hours [3], but does this situation change when remifentanil is given for days rather than hours? One study looked at the pharmacodynamic offset of remifentanil at various time points over 3 days in ICU patients and found that the offset time was constant over this period [5]. In the present study there was no evidence of accumulation of remifentanil over time. Of the patients who were not extubated within 10 days, on discontinuing the remifentanil infusion the mean time to offset of pharmacodynamic effect was only 15 min and was identical to that obtained in the study above [5]. A recent paper has shown that if sedation in ICU patients was stopped on a daily basis, it considerably reduced the duration of mechanical ventilation and ICU stay [23]. However, these patients were heavily sedated. This study and the above study demonstrates that if patients are sedated to an optimum SAS and PI score with remifentanil, then despite the duration of sedation the pharmacodynamic offset time is about 15 min, making a daily 'wake-up call' unnecessary for the reasons of decreasing the amount of total sedation and aiding extubation and ICU discharge.

The duration for which patients had an optimum SAS and PI score was the same in each group and was more than 96% of the duration for which patients were receiving treatment. This is to be expected, because the aim of the study was to titrate the analgesic/sedative regimes to achieve target SAS and PI scores that were optimal. Thus, the only effect on the primary endpoint was the drugs used and not the variation in sedation/analgesic levels.

### Safety

The reported incidence of adverse effects in this study was the same in the remifentanil and comparator groups. The incidence was also similar to those in other studies looking at the safety of remifentanil and comparator agents in ICU patients [5,11,12]. The adverse events were also in keeping with events that one would expect in an ICU population. The only serious adverse event that was reported as drug-related occurred in the comparator group. This was of severe life-threatening hypotension. Deaths were the same in each group and at the expected rate for an ICU population. No deaths were reported as a result of the study drug. There were no reports of muscle rigidity or of prolonged μ-opioid effects as a consequence of using remifentanil. There was no statistical difference in the incidence of re-intubation between the two treatment groups.

There was no evidence of adverse SAS or PI scores on discontinuing the remifentanil. The mean SAS and PI scores were the same in each group and stable for the 24-hour post-treatment period. Investigators were able to use the regime of their choice to optimise PI and SAS after extubation, in line with routine clinical practice. There was no evidence that remifentanil sensitised opioid receptors or that the rapid offset of pharmacodynamic effects of remifentanil caused problems with control of pain after extubation.

### Exposure to study drugs

In the comparator group the weighted mean infusion rates of fentanyl and morphine were relatively constant throughout the study. The large increase in the morphine requirements on days 8 and 9 represented one patient only.

The weighted mean remifentanil infusion rate to maintain optimum SAS and PI scores was 19.2 μg kg^-1 ^h^-1 ^(0.32 μg kg^-1 ^min^-1^) (Table 4). This is slightly higher than reported previously, but well within the infusion rates recommended [5,11]. The remifentanil use remained relatively constant throughout the study, and generally the remifentanil was given for longer than the other opioids (Fig. 5, Table 4).

The midazolam requirements were considerably reduced in the remifentanil group and were relatively constant throughout the treatment period (Figs 4 and 6). This is a reflection of the hypnotic-agent-sparing effects of remifentanil and the ease with which the infusion can be titrated to obtain optimum patient comfort [11,17].

In contrast, the mean total daily requirements for midazolam in the comparator group varied considerably throughout the study period to maintain an optimal SAS for 97% of the time. There was an up to 10-fold difference in mean daily midazolam requirements in the fentanyl subgroup (226 mg on day 4; 26 mg on day 10) and a sevenfold difference in the morphine subgroup (189.6 mg on day 5; 28.4 mg on day 10). This accounts for the difference in mean total midazolam requirements between the groups. The requirements for midazolam peaked between days 3 and 5 in the comparator groups, and then tailed off (Fig. 6). The above observations are a reflection of two factors. First, midazolam was used as the primary hypnotic agent in the comparator group and it was therefore adjusted first. Second, although the opioid infusion rates remained relatively constant, as the study progressed, opioid and midazolam accumulation occurred. The accumulation, with large body stores of these drugs, contributed to the sedation in these patients. Therefore the requirement for midazolam administration to maintain a constant SAS and PI score tailed off after a certain period. The context-sensitive half-time of the comparator agents is known to increase with time, and the comparator agent's elimination is organ dependent [1,24].

There was no clinical evidence of the development of tolerance to remifentanil as demonstrated by escalating remifentanil requirements or post-infusion opioid requirements. The weighted mean infusion rate of remifentanil increased slightly until day 3, and was then constant until day 10. The SAS and PI scores at the start and throughout the post-treatment period were the same as in the comparator, and were constant.

There was a group of patients who received only midazolam to provide patient comfort during their stay in the ICU. This occurred at one hospital where this was the preferred practice for the treatment of medical patients without injuries and reflected normal clinical practice at that site.

The present study compares two different sedation techniques. One technique used the analgesic component of the regime as the main variant for sedation (remifentanil). The other used the hypnotic component as the main variant for sedation (midazolam). Although this second group gave rise to three subgroups because either fentanyl, morphine or no analgesic was used, this was unimportant. The main aim was to compare the remifentanil regime with common 'standard practice' for sedation in ICU patients. It would not have been representative of clinical practice to compare two opioids in an analgesia-based regime: the commonly used opioids cannot be used in this way because of the real risk of accumulation. The present study clearly demonstrates that the remifentanil-based regime is superior in terms of reduced time for weaning and, more importantly, reduced duration of mechanical ventilation.

## Conclusion

The use of remifentanil in an analgesic-based sedative regime in critically ill patients significantly decreases the duration of mechanical ventilation and of weaning. It is sedative sparing and has a very rapid offset even after a 10-day infusion, with no evidence of accumulation. Remifentanil was well tolerated throughout a 10-day infusion. The adverse event profile was similar in remifentanil-based and hypnotic-based regimes. No adverse events relating to muscle rigidity or prolonged μ-opioid effects were reported. The SAS and PI scores after treatment were comparable. There was no evidence of the development of tolerance to remifentanil and there was no difficulty in maintaining optimal SAS and PI scores after treatment with remifentanil with the use of standard treatment regimes.

## Key messages

• 	The use of remifentanil-based analgesia and sedation significantly reduced the duration of mechanical ventilation.

• 	Weaning patients from mechanical ventilation can be achieved significantly faster with remifentanil-based analgesia and sedation.

• 	Remifentanil has a very rapid offset even after a 10-day infusion with no evidence of accumulation.

• 	There was no evidence of the development of tolerance to remifentanil and there was no increase in opioid requirements after treatment with remifentanil, even after prolonged use.

• 	A remifentanil-based analgesia and sedation regimen is well tolerated when used for up to 10 days in critically ill patients requiring mechanical ventilation and has a safety profile similar to that observed for hypnotic-based sedation.

## Abbreviations

HR = heart rate; ICU = intensive care unit; MAP = mean arterial pressure; PI = pain intensity; SAS = Sedation–Agitation Scale.

## Competing interests

DB has no competing interests. AK, MM, RM, SA and I-LJ received payment from GlaxoSmithKline (either personally or to their respective department) depending on the number of patients recruited. PP and AJTK are employees of GlaxoSmithKline.

## Authors' contributions

DB made substantial contributions to the conception, design and interpretation of the data collected in this study, and drafted the manuscript. AK, MM, RM SA and I-LJ performed the study and provided critical review of the manuscript. PP coordinated the development and conduct of the study. AJTK contributed to the design of the study and the interpretation of the data and provided critical review of the manuscript. All authors read and approved the manuscript.

## Supplementary Material

Additional File 1 A Word file showing the definitions of the scores on the Sedation–Agitation Scale. Click here for file

Additional File 2 A Word file showing the definitions of pain intensity scores. Click here for file

## Figures and Tables

**Table 1:** Patient characteristics and baseline clinical assessments

Characteristic	Remifentanil	Comparator
Number of patients treated	57	48
Medical (%)/post-surgical (%)	49 (88)/7 (13)	44 (92)/4 (8)
Emergency (%)/elective (%)	27 (84)/5 (16), *n *= 32	21 (91)/2 (9), *n *= 23
Age (years)	52.2 ± 18.4	57.3 ± 18.1
Male (%)/female (%)	39 (68)/18 (32)	32 (67)/16 (33)
Height (cm)	171.2 ± 9.7	169.0 ± 7.9
Weight (kg)	78.6 ± 13.41	76.3 ± 15.50
SAPS II on admission	43.0 ± 15.6	43.3 ± 11.2
MAP (mmHg)^a^	88.8 ± 16.5	88.9 ± 14.8
Heart rate (b.p.m.)^a^	98.9 ± 20.1	95.9 ± 15.5
SAS score^a^	3.3 ± 1.3	3.3 ± 1.4
PI score^a^	2.0 ± 1.2	2.1 ± 1.1

^a^Baseline values. Where errors are given, results are means± SD. MAP, mean arterial pressure; PI, pain intensity; SAS, Sedation–Agitation Scale.

**Table 2:** Study endpoints

Characteristic	Remifentanil (*n *= 57)	Comparator (*n *= 48)	*P*
Number (%) of patients extubated	29 (51%)	16 (33%)	
Time from start of study drugs to weaning (h)	83.0	98.0	0.523
Difference (95% CI)	-15.0 (-61.8 to 31.8)		
Time from start of study drugs to extubation(h)	94.0	147.5	0.033
Difference (95% CI)	-53.5 (-111.4 to 4.4)		
Time from weaning time until extubation (h)	0.9	27.5	<0.001
Difference (95% CI)	-26.6 (-40.8 to -12.4)		
Time from start of study drugs until ICU discharge (h)	187.3	209.8	0.326
Difference (95% CI)	-22.5 (-201.5 to 156.5)		

Point estimates are 75th centiles. CI, confidence interval.

**Table 3:** Adverse event profile

Characteristic	Number of patients (%)	
	
	Remifentanil (*n *= 57)	Comparator (*n *= 48)
Any adverse event	19 (33)	16 (33)
Any drug-related adverse event	6 (11)	4 (8)
Any serious adverse event	7 (12)	6 (13)
Any drug-related serious adverse event	0 (0)	1 (2)
Premature discontinuation from the study	12 (21)	10 (21)
Deaths	7 (13)	5 (10)
Re-intubated within 10 days^a^	7 (25)	2 (12)
Most commonly occurring adverse events (≥ 5%)		
Hypotension	3 (5)	4 (8)
Atrial fibrillation	4 (7)	2 (4)
Vomiting	3 (5)	0 (0)
Septic shock	0 (0)	3 (6)

^a^*P *value for continuity-corrected χ^2^. 95% confidence interval -4 to 23. *P *= 0.193. Time to re-intubation ranged from 2 h to 3 days after stopping remifentanil, and 7 hours to 3 days after stopping comparator agent.

**Table 4:** Exposure to study drug for all patients treated

Parameter	Remifentanil (*n *= 57)	Fentanyl (*n *= 30)	Morphine (*n *= 7)
Mean duration of infusion (h)	147.2	126.4	120.5
Mean total opioid dose	221,614 μg	20,702 μg	237.5 mg
Mean weighted mean opioid infusion rate(SD)	19.2^a ^(12.2) μg kg^-1 ^h^-1^	3.0 (3.35) μg kg^-1 ^h^-1^	0.042 (0.028) mg kg^-1 ^h^-1^

^a^Equivalent to 0.32 μg kg^-1 ^min^-1^.
