# Supplementary material for: Decreased duration of mechanical ventilation when comparing analgesia-based sedation using remifentanil with standard hypnotic-based sedation for up to 10 days in intensive care unit patients: a randomised trial [ISRCTN47583497]
Source: Crit Care. 2005 Mar 15;9(3):R200–10. doi: 10.1186/cc3495 (PMC1175879; doi:10.1186/cc3495)
Supplement: Additional File 1 — A Word file showing the definitions of the scores on the Sedation–Agitation Scale. [file cc3495-S1.doc]

**Additional file 1**

**The Sedation–Agitation Scale (SAS) [18]**

| **Score** | **Description** | **Example** |
| --- | --- | --- |
| 7 | Dangerous agitation | Pulling at endotracheal tube, trying to remove catheters, climbing over bedrails, thrashing from side to side, striking at staff |
| 6 | Very agitated | Patient does not calm down to verbal instructions or reassurance, requires physical restraint, biting endotracheal tube |
| 5 | Agitated | Anxious or agitated but calms down to verbal instructions or reassurance |
| 4 | Calm, co-operative | Calm, easily rousable, follows commands |
| 3 | Sedated | Difficult to rouse, awakens to verbal stimuli or gentle shaking but drifts off again, will follow simple commands |
| 2 | Very sedated | Can be roused by physical stimuli but does not communicate or follow commands, may move spontaneously |
| 1 | Not rousable | May move or grimace minimally to stimuli but does not communicate or follow commands |
