# Supplementary material for: Decreased duration of mechanical ventilation when comparing analgesia-based sedation using remifentanil with standard hypnotic-based sedation for up to 10 days in intensive care unit patients: a randomised trial [ISRCTN47583497]
Source: Crit Care. 2005 Mar 15;9(3):R200–10. doi: 10.1186/cc3495 (PMC1175879; doi:10.1186/cc3495)
Supplement: Additional File 2 — A Word file showing the definitions of pain intensity scores. [file cc3495-S2.doc]

**Additional file 2**

**Pain intensity score**

The assessment of pain intensity will be made by the nurse responsible for caring for the patient. The following six point pain intensity scale will be used during this study:

1 = No Pain

2 = Mild Pain

3 = Moderate Pain

4 = Severe Pain

5 = Very Severe Pain

6 = Worst Possible Pain
